# Anti-PTSD Effects of Hypidone Hydrochloride (YL-0919): A Novel Combined Selective 5-HT Reuptake Inhibitor/5-HT_1A_ Receptor Partial Agonist/5-HT_6_ Receptor Full Agonist

**DOI:** 10.3389/fphar.2021.625547

**Published:** 2021-02-10

**Authors:** Wen-Gang Liu, Li-Ming Zhang, Jun-Qi Yao, Yong-Yu Yin, Xiao-Ying Zhang, Yun-Feng Li, Jiang-Bei Cao

**Affiliations:** ^1^ Medical School of Chinese PLA, Beijing, China; ^2^ Department of Anesthesiology, the First Medical Center, Chinese PLA General Hospital, Beijing, China; ^3^ Beijing Institute of Pharmacology and Toxicology, State Key Laboratory of Toxicology and Medical Countermeasures, Beijing Key Laboratory of Neuropsychopharmacology, Beijing, China; ^4^ Beijing Institute of Basic Medical Sciences, Beijing, China

**Keywords:** hypidone hydrochloride (YL-0919), posttraumatic stress disorder, cognition impairment, brain-derived neurotrophic factor, neuroplasticity

## Abstract

Posttraumatic stress disorder (PTSD) is a debilitating trauma and stressor-related disorder that has become a major neuropsychiatric problem, leading to substantial disruptions in individual health and societal costs. Our previous studies have demonstrated that hypidone hydrochloride (YL-0919), a novel combined selective 5-HT reuptake inhibitor/5-HT_1A_ receptor partial agonist/5-HT_6_ receptor full agonist, exerts notable antidepressant- and anxiolytic-like as well as procognitive effects. However, whether YL-0919 exerts anti-PTSD effects and its underlying mechanisms are still unclear. In the present study, we showed that repeated treatment with YL-0919 caused significant suppression of contextual fear, enhanced anxiety and cognitive dysfunction induced by the time-dependent sensitization (TDS) procedure in rats and by inescapable electric foot-shock in a mouse model of PTSD. Furthermore, we found that repeated treatment with YL-0919 significantly reversed the accompanying decreased expression of the brain-derived neurotrophic factor (BDNF) and the synaptic proteins (synapsin1 and GluA1), and ameliorated the neuroplasticity disruption in the prefrontal cortex (PFC), including the dendritic complexity and spine density of pyramidal neurons. Taken together, the current study indicated that YL-0919 exerts clear anti-PTSD effects, which might be partially mediated by ameliorating the structural neuroplasticity by increasing the expression of BDNF and the formation of synaptic proteins in the PFC.

## Introduction

Posttraumatic stress disorder (PTSD) is characterized by intrusive re-experiences of trauma-related events, sustained avoidance of stimuli related to traumatic events, negative changes in cognition and mood, exaggerated startle responses and hypervigilance according to the Diagnostic and Statistical Manual of Mental Disorders 5th edition (DSM-5). Epidemiological studies have revealed that the lifetime prevalence of PTSD ranges from 1.3 to 12.2% in the populations studied (Shalev et al., 2017). In addition, it is common that PTSD cooccurs with major depressive disorder, substance abuse and other anxiety disorders, and these comorbidity conditions will further worsen the severity of PTSD, leading to a high risk of a suicide attempt and causing substantial disruptions for the individual and in society (Sareen, 2014; Shalev et al., 2017).

Although considerable progress has been made in research on the mechanisms and pathophysiology research of PTSD, no breakthrough medications that treat symptoms or improve prognosis in PTSD patients have been discovered. To data, only paroxetine and sertraline, two selective serotonin reuptake inhibitor (SSRI) antidepressants, have been approved by the Food and Drug Administration (FDA) as the first-line pharmacotherapeutic agents for the PTSD treatment. Unfortunately, some drawbacks of SSRIs, including a delayed onset of action, partial response, cognitive dysfunction, and undesirable side effects such as sexual dysfunction, limit their clinical application (Krystal et al., 2017). Therefore, great efforts have been made to search for the better agents for the efficacious treatment of PTSD.

Accumulating evidence has shown that serotonin (5-HT) system dysfunction plays a critical role in the neuropathology and treatment of PTSD. Genetic deletion of 5- HT in the mouse brain has been linked to enhanced contextual fear conditioning and expression, and the disruptions of hippocampal synaptic plasticity caused by foot-shock exposure is restored by application of 5-HT in 5-HT-deficient mice (Dai et al., 2008). It was also reported that 5-HT transporter knockout mice show enhanced anxiety-like behaviors and exaggerated stress responses after predator stress (Holmes et al., 2003). Moreover, the amelioration of symptoms following SSRI treatment has increased attention to the role of 5-HT in the neurobiology and treatment of PTSD.

During the last decade, 5-HT_1A_ receptors have been implicated as pivotal targets for PTSD. Activation of postsynaptic and, to a less extent, presynaptic 5-HT_1A_ receptors results in inhibition of the acquisition and expression of contextual fear (Homberg, 2012). Furthermore, 5-HT_1A_ agonists can not only regulate conditioned fear but also regulate the effects of SSRIs. For example, cotreatment with a 5-HT_1A_ receptor agonist enhanced the inhibition of the contextual fear expression induced by SSRI (Nishikawa et al., 2007). Moreover, enhanced anxiety and increased fear response were also found in 5-HT_lA_ receptor knockout mice (both autoreceptors and heteroreceptors) (Ramboz et al., 1998; Klemenhagen et al., 2006). Conditional tissue-specific rescues have further suggested a critical role of postsynaptic 5-HT_1A_ receptors in the forebrain in anxiety-like behaviors. Particularly, overexpression of 5-HT_1A_ receptors in forebrain areas such as the cortex and striatum (in the absence of autoreceptors) restored the increased anxiety behavior in 5-HT_1A_ knockout mice (Gross et al., 2002). It was also reported that knockout of endogenous 5-HT_1A_ autoreceptors was sufficient to increase anxiety in the adult mice (Richardson-Jones et al., 2011).

A considerable number of studies have shown that PTSD is associated with significant impairments in cognitive functioning (Vasterling et al., 1998), particularly in terms of attention, verbal learning and verbal memory. Moreover, these cognitive deficits have been shown to cause adverse effects in the context psychotherapy and for functional prognoses in individuals with PTSD (Falconer et al., 2013). Decades of research have demonstrated the role of the 5-HT system involved in the processes of learning and memory, and the 5-HT_6_ receptors have become a hopeful target for the treatment of cognitive dysfunction. Indeed, 5-HT_6_ receptor agonists have been found to dramatically improve cognitive performance in different learning and memory tests performed with animals and humans (Kendall et al., 2011; Rychtyk et al., 2019). Furthermore, a behavioral study revealed that selective 5-HT_6_ receptor agonists could exert significant anti-anxiety effects in rats (Carr et al., 2011).

Our previous studies have shown that hypidone hydrochloride (YL-0919) [(1-(1-benzyl-4-hydroxypiperidin-4-ylmethyl)-2(1H)-pyridinone hydrochloride], a novel triple-target-directed antidepressant manifesting as an SSRI, a 5-HT_1A_ receptor partial agonist and a 5-HT_6_ receptor full agonist (Chen et al., 2013; Chen et al., 2018), exerts notable antidepressant, anxiolytic-like and procognitive behavioral effects (Ran et al., 2017; Chen et al., 2018)without producing sexual dysfunction (Zhang et al., 2017). Our recent study found that YL-0919 may produce a fast-acting effect indicated by rapidly influencing the synaptic plasticity [enhancing long-term potentiation (LTP)] in the hippocampus as well as increasing the synaptic proteins and dendritic complexity in the prefrontal cortex (PFC) of rats (Zhang et al., 2017; Sun et al., 2019).

In the present study, we intended to explore whether the triple-targeted drug YL-0919 could exert a therapeutic effect in two rodent models of PTSD. First, we determined the effects of YL-0919 in alleviating PTSD-like behaviors, including enhanced fear responses and anxiety, as well as cognitive impairment in the time-dependent sensitization (TDS)-induced rat model of PTSD and a mouse model of PTSD involving inescapable electric foot shock (IFS). Furthermore, the changes in synaptic proteins as well as neuroplasticity in the PFC after chronic YL-0919 administration were also assessed in IFS-exposed mice.

## Materials and Methods

### Animals

Both 62 eight-week-old male Sprague–Dawley rats (weighing 180–200 g, used for TDS procedure) and 52 eight-week-old male C57BL/6 mice (weighing 21–23 g, used in the electric foot-shock procedure) were purchased from Beijing SPF Biotechnology (Beijing, China). All animals were randomly assigned to each group with 10–11 animals in each group. The animals were housed in standard cages with free access to water and food. All animals were acclimatized to temperature (22 ± 1 °C), humidity (55 ± 10%) and light (12 h light-dark cycle with lights on 7:00 AM) controlled environmental conditions for 7 days before experimentation. The experimental procedures were approved by the Institutional Animal Care and Use Committee, and all experiments were performed in line with the National Institutes of Health Guide for the Care and Use of Laboratory Animals. All efforts were made to minimize animal suffering.

### Drugs and Reagents

YL-0919 (white powder, purity ≥ 99.8%) was synthesized at our institute. Sertraline (Ser) was purchased from Sigma-Aldrich (St Louis, MO, United states). Both YL-0919 (0.625, 1.25 or 2.5 mg/kg) and Ser (15 mg/kg) were dissolved in distilled water and administered by intragastric gavage (i.g.) at volumes of 2 ml/kg (rats) or 10 ml/kg (mice). Drugs treatment started from the first day of the TDS procedure in rats and from the first day after the IFS procedure in mice, and both administrations continued through the end of the experiments. The dosages of YL-0919 selected in experiments were based on the previous behavioral tests (Ran et al., 2017; Zhang et al., 2017; Sun et al., 2019).

### Behavioral Experiments

#### Long-Term Behavioral Effects of YL-0919 After TDS Procedures in Rats

The TDS procedure was conducted as previously described (Zhang et al., 2014). Briefly, after 1 week of acclimatization, each rat was immobilized inside a polyethylene restraint apparatus for 2 h on the first day. The rats were held in a state of complete immobility by adjusting the size of the restraint apparatus. After the restraint period, the rats were forced to individually swim for 20 min in an acrylic cylinder (25 cm diameter, 60 cm height) filled with water (24 °C) to 2/3 of its height. Following a 15-min recovery, the rats were then exposed to diethyl ether until loss of consciousness. The rats were left undisturbed for a week to recover and then re-exposed to a brief stressor (20-min swim stress) on day 7. The control rats were subjected to handling twice for several minutes each time.

**FIGURE 1:**
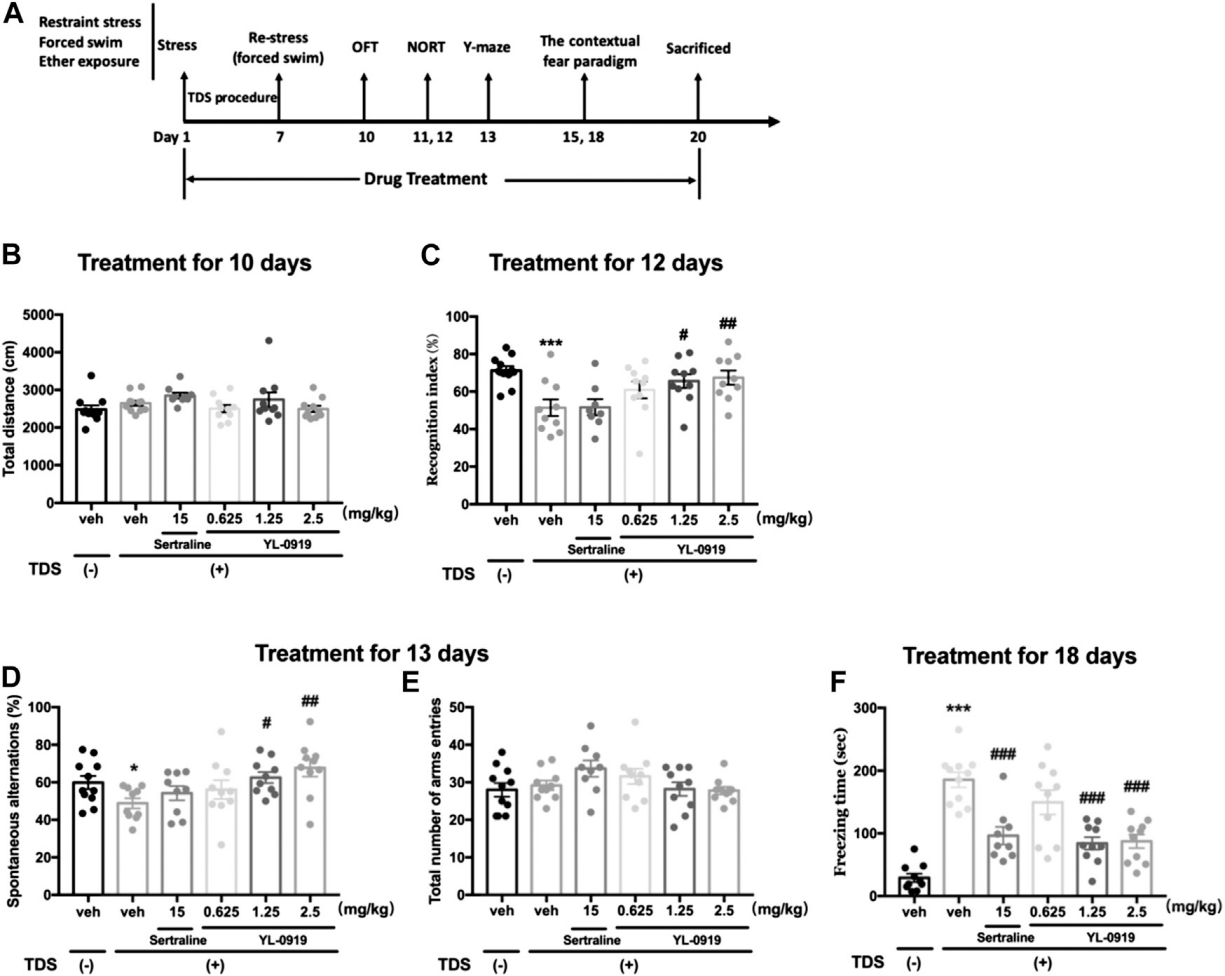
Long-term behavioral effects of repeated administration of Ser or YL-0919 in post-TDS rats. Experimental timeline and treatment schedule for the TDS-induced rat model of PTSD **(A)**. The TDS procedure and the repeated drug treatments did not significantly affect the total distance moved **(B)**. Post-TDS rats exhibited significant reductions in both recognition index and spontaneous alternations in the NORT and Y-maze test, respectively. Repeated administration of YL-0919 (1.25 and 2.5 mg/kg) significantly ameliorated the decrease in both the recognition index **(C)** and percentage of spontaneous alternations **(D)** on day 12 and day 13, respectively. In contrast, the recognition index and the spontaneous alterations were not reversed by Ser treatment in TDS rats. The TDS procedure and the repeated drug treatments did not significantly affect the total number of arm entries between each group in the Y-maze test **(E)**. Rats exposed to the TDS procedure showed a significant increase in contextual freezing time. Repeated administration of Ser or YL-0919 (1.25 and 2.5 mg/kg) significantly reduced contextual freezing behavior on day 18 **(F)**. The data shown are the means ± SEM; *n* = 9–11; *p < 0.05, ***p < 0.001 vs. the vehicle-treated TDS (−) group; ^#^ *p* < 0.05, ^##^ *p* < 0.01, ^###^ *p* < 0.001 vs. the vehicle-treated TDS (+) group.

From the first day of the TDS procedure, Ser (15 mg/kg), YL-0919 (0.625, 1.25, and 2.5 mg/kg) or vehicle was administered (i.g.) once a day between 8:00–9:00 and continued through the end of the experiment. On days 10–18, the rats were assessed using different behavioral paradigms, including the open field test (OFT), novel object recognition test (NORT), spontaneous alternation test in the Y-maze and contextual fear paradigm 1 h after drug administration. The diagram of the treatment schedule and the order of behavioral tests are presented in Figure 1A.

##### Open Field Test

To examine whether the alleviation of PTSD-like behaviors by YL-0919 depended on altered locomotor activity, we investigated the level of spontaneous locomotor activity using the OFT as previously described with minor modifications (Zhang et al., 2014). The rats were placed in the corner of a plastic chamber (76 × 76 × 46 cm) and were allowed to freely move for 5 min; the total distance that the rats traveled was recorded and analyzed automatically by the SMART Video-Tracking System V3.0 (Panlab, Barcelona, Spain).

##### Novel Object Recognition Test

The NORT is an extremely validated task for recognition memory and was performed as previously described with minor modifications (de Lima et al., 2005). The NORT consisted of three consecutive periods: habituation period, acquisition period and recall period. Briefly, the rats were free to move in an open-field box (60 × 60 × 60 cm) for 5 min to acclimate to the environment. On the following day, acquisition training was conducted by placing each rat into the experimental apparatus for 5 min, and two identical objects (green Erlenmeyer flasks) were located in symmetric positions, 10 cm from the walls. On the second day of acquisition training, the rats were tested for memory recall for 5 min in the same apparatus except that one of the familiar objects was replaced with a novel object (i.e., orange square bottle). The exploration times with the familiar object (T_f_) and the novel object (T_n_) were measured. The recognition index (RI) was calculated and represented the ratio T_n_/(T_f_ + T_n_). Exploratory behavior was defined as touching, sniffing or facing the object within 2 cm. After each trial, the objects and box were thoroughly cleaned with 30% ethanol to remove odor cues.

##### Spontaneous Alternation Test in the Y-Maze

The Y-maze test was carried out as previously described (Rychtyk et al., 2019). The Y-maze was a Y-shaped experimental apparatus made of three plastic arms with an angle of 120° between each arm (60 × 10 × 20 cm). The three arms were marked A, B, and C. Each rat was placed at the end of one arm and allowed to freely move in the Y-maze for 8 min. Spontaneous alternation behavior, defined as consecutive entries into all the three arms in overlapping triplet sets (such as ABC, BCA, and CAB), was videotaped by a digital camera and automatically analyzed by the SMART software. The percentage of spontaneous alternations was calculated using the following formula [(number of alternations)/(total number of arm entries - 2)] × 100.

##### Contextual Fear Paradigm

The experimental procedure was performed as previously described on day 15 after the TDS procedure (Zhang et al., 2014). Each rat was exposed to the conditioning context in the conditioning chamber without any stimulus for 180 s, followed immediately by a foot shock (0.8 mA, 4 s) delivered through a stainless-steel grid floor (Med Associates Inc., Georgia, VT, United States). Three days after the initial foot shock (on day 18), the rat was returned to the same conditioning chamber without foot shock for 5 min, and the freezing time (complete immobility with the exception of breathing) that reflect the contextual fear response was recorded and automatically analyzed by Video Freeze software (Med Associates Inc., Georgia, VT, United States).

#### Long-Term Behavioral Effects of YL-0919 After Inescapable Electric Foot-Shock Procedures in Mice

Further another set of experiments were performed to explore anti-PTSD effects of the YL-0919 in the inescapable electric foot-shock mouse model of PTSD. The experimental procedure was conducted as previously described with minor modifications (Zhang et al., 2012). The mice were transferred into the testing room at least 1 h before the experiment started to minimize possible disturbances of cage transportation on behavioral testing and to acclimate animals to the experimental conditions. During the training session, a plexiglass box (20 × 10 × 10 cm) with a stainless-steel grid floor (4-mm diameter, 9-mm interval) was used. Briefly, after a 5-min acclimation period, each mouse was exposed to 15 intermittent and inescapable electric foot-shocks transmitted through the grid floor by a separate electric shock generator (intensity: 0.8 mA, interval: 10 s, and duration: 10 s) (Med Associates Inc., Georgia, VT, United States) for two consecutive days. The control mice were exposed to the identical chamber without electric foot shocks for 10 min.

**FIGURE 2:**
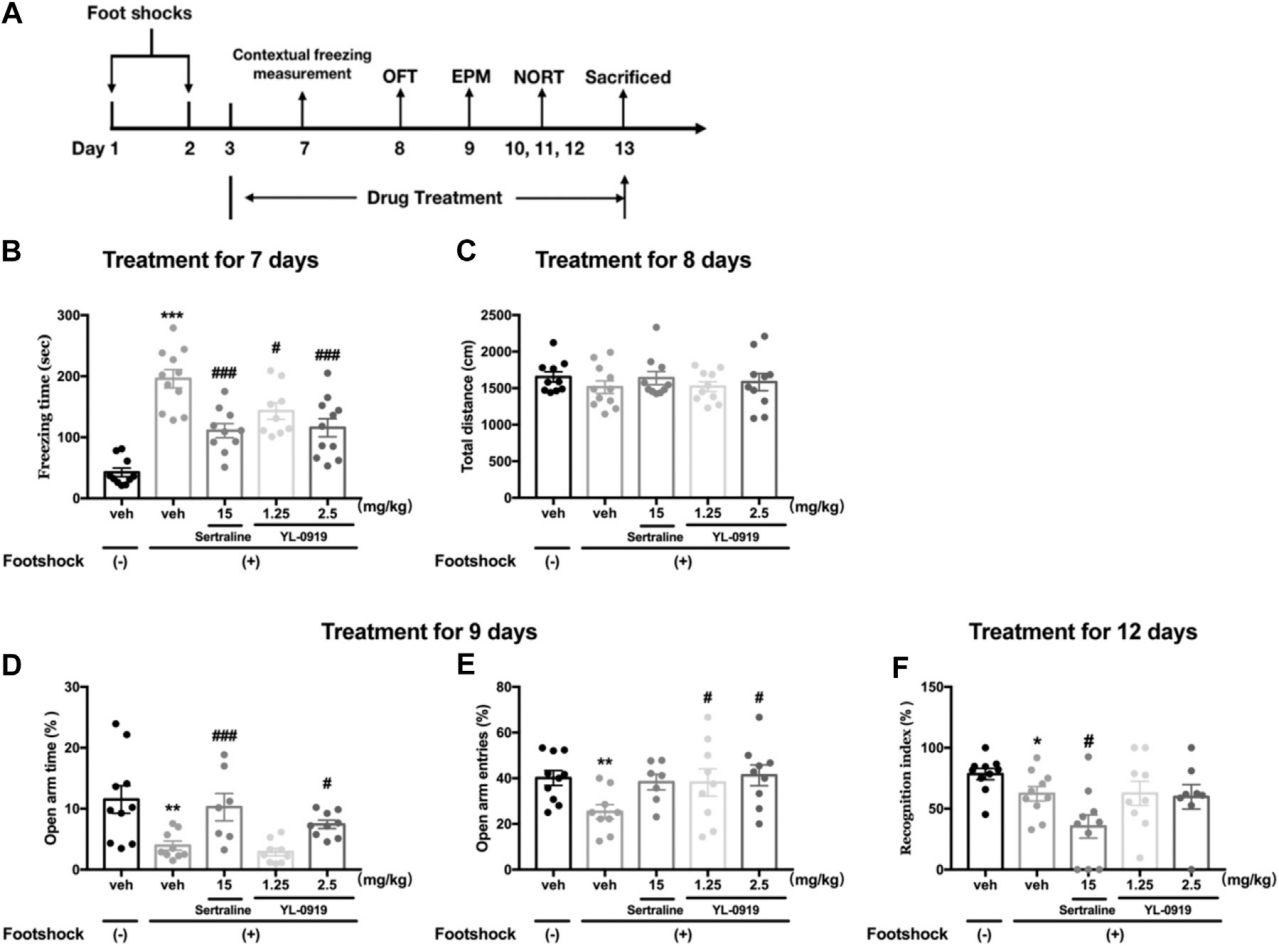
The effects of repeated administration of Ser or YL-0919 on PTSD-like behavior in IFS-exposed mice. Experimental timeline and treatment schedule for the IFS-exposed mice **(A)**. The post-IFS mice showed a significant increase in contextual freezing time, decreased percentages of open-arm entries and open-arm duration times in the elevated plus maze test, and a decreased recognition index in the novel object recognition test. Repeated treatment with YL-0919 (1.25 and 2.5 mg/kg) or Ser (15 mg/kg) ameliorated the contextual fear response on day 7 **(B)**. YL-0919 (2.5 mg/kg) or Ser (15 mg/kg) reversed the decreased percentages of open-arm duration time **(D)**, and YL-0919 (1.25 and 2.5 mg/kg) reversed the decreased percentages of open-arm entries in the EPM test on day 9 **(E)**. However, sertraline further decreased the recognition index, and YL-0919 had no effect on the decreased recognition index **(F)**. The IFS procedure and the repeated drug treatments did not significantly affect the total distance moved **(C)**. The data shown are the means ± SEM; *n* = 7–11; *p < 0.05, **p < 0.01, ***p < 0.001 vs. the vehicle-treated foot-shock (−) group; ^#^ *p* < 0.05, ^###^ *p* < 0.001 vs. the vehicle-treated foot-shock (+) group.

From the first day after the IFS procedure, Ser (15 mg/kg), YL-0919 (1.25 and 2.5 mg/kg) or vehicle was administered (i.g.) once a day between 8:00–9:00 and continued through the end of the experiment. After the 2-days IFS procedure, behavioral tests including the measurement of contextual freezing behavior, OFT, elevated plus maze (EPM) test and NORT were performed on sequential days. All behavioral tests were conducted 1 h after the drug or vehicle administration. The flow chart of the treatment schedule and behavioral tests are presented in Figure 2A.

##### Contextual Freezing Measurement

Freezing behavior during re-exposure to the same shock environment is a response to trauma-related cues and is measured as an index of associative conditioned fear memory, reflecting the symptoms of PTSD (Siegmund and Wotjak, 2007). The test was conducted as previously described with minor modifications (Zhang et al., 2012). All mice were returned to the same conditioning chamber (as a situational reminder) without foot shocks for 5 min on day 7. The duration of freezing behavior of the mice was recorded and automatically analyzed by Video Freeze software.

##### Open Field Test

Spontaneous locomotor activity was measured using the OFT on day 8, which referred to the procedure used in the rat experiment. The mice were placed in the corner of a plastic box (40 × 40 × 20 cm), and the spontaneous locomotor of the mice was videotaped for 5 min. Then, the total distance traveled by the mice was automatically analyzed by SMART software.

##### Elevated Plus Maze Test

The EPM test has been well validated for assessing PTSD-associated anxiety-like behavior in rodents (Walf and Frye, 2007; Zhang et al., 2012). The plastic apparatus was composed of four branching arms (30 × 30 cm), including two open arms and two enclosed arms. The four arms were connected by a center platform (8 × 8 cm), and the whole apparatus was 50 cm above the ground. The mouse was placed in the center of the platform facing an enclosed arm and was free to move for 5 min in the maze. The number of entries into and time spent in each arm (open and closed) were measured following the criterion of all four paws crossing the dividing line. For analysis purposes, the following formulas were used to calculate the percentages of open-arms entries and the percentages of open-arms duration time: the open-arm entries/total entries into four arms and open-arm duration time/the total duration time in four arms.

##### Novel Object Recognition Test

NORT was conducted as previously described and was similar to the procedure used in the rat experiment. During habituation, each mouse was allowed to freely explore the environment in a square open field (40 cm × 40 cm × 20 cm) for 5 min. In the acquisition period after 24 h, each mouse was placed into the open field containing the same two fixed black wooden spheres and was free to explore the environment for 5 min. On the second day of acquisition training, the mice were again placed in the same apparatus for 5 min to test for memory recall, except that one of the familiar objects was replaced with a novel object (white wooden cube), and the exploration time of the familiar object (T_f_) and the novel object (T_n_) were measured. The RI was calculated according to the formula used for the NORT with rats.

### Western Blotting

Four mice per group were sacrificed after the electric foot-shock procedures were sacrificed, and carefully dissected to remove the PFC. The separated prefrontal cortical tissues were homogenized by RIPA lysis buffer (Millipore, Billerica, MA, United States) containing protease inhibitor and phosphatase inhibitor cocktail (Thermo Pierce, Rockford, IL, United States). The dissolved proteins were centrifuged at 10,000×g for 10 min at 4 °C, and then the supernatants were collected for further detection. Samples containing 50 μg protein were resolved by electrophoresis, transferred to PVDF membranes (Millipore, Billerica, MA, United States) and blocked with 5% skimmed milk in TBST. The membranes were incubated with primary antibodies against synapsin-1 (1:1,000; Cell Signaling Technology, Danvers, MA, United States), GluA1 (1:1,000; Cell Signaling Technology, Danvers, MA, United States), BDNF (1:1,000; Abcam, Cambridge, United Kingdom) or *β*-actin (1:300; Santa Cruz Biotechnology, Santa Cruz, CA, United States) at 4 °C overnight. The membranes were washed with TBST for 5 min, 3 times. Then the membranes were incubated for 1 h at room temperature in fluorescence secondary antibodies (1:10,000, IRDye 800-conjugated affinity purified goat anti-rabbit or IRDye 700-conjugated affinity purified goat anti-mouse, LI-COR Biosciences). The specific bands were detected using the Odyssey infrared imaging system. The relative levels of the respective proteins were expressed by the density ratio of the target protein band to the *β*-actin band, and the relative values were normalized to the vehicle-treated control group for each protein.

### Golgi-Cox Staining

The Golgi-Cox staining test was performed according to the manufacturer’s instructions (FD Neuro Technologies, Ellicott City, MD, United States). Briefly, the brain tissues of mice were quickly removed from the skull and cleaned with distilled water. Subsequently, brains were placed into the impregnation solution containing solutions A and B for immersion. The samples were kept at room temperature in the dark for 2 weeks. Three days after transferring into solution C (at room temperature in the dark), coronal sections (100 μm) of the PFC were cut on a cryostat (CM1860 UV, Leica Microsystem Ltd., Wetzlar, Germany) at −22 °C. Sections were mounted on gelatin-coated microscope slides with solution C and dried at room temperature in the dark. Then, sections were rinsed and placed in the working solution containing solutions D and E for staining followed by dehydration in gradient ethanol. Ultimately, the sections were cleared in xylene and mounted with neutral resin.

Qualifying PFC neurons were photographed with the Nikon A1 HD25 confocal microscope (Nikon Corporation, Tokyo, Japan). The criterion applied to select neurons for reconstruction has been well previously described (Flores et al., 2005). Briefly, only well impregnated and clearly distinguishable pyramidal neurons with unbroken dendrites were chosen in the present study. Neurons were traced and digitally reconstructed using ImageJ software. The total dendritic length and the total number of dendritic branches, reflecting dendritic complexity, were analyzed. Dendritic spines were photographed under a high power (1,000 ×) microscope (DM4000B, Leica Microsystem Ltd., Wetzlar, Germany). The entire, most distal branch of a dendrite (over 20 μm in length) was included in the study. Spines were counted along the length of the dendritic branch by the ImageJ software, and the spine density results were expressed as spines/10 μm. For the quantitative analysis, two to three neurons from each section, three sections from each animal, and four animals per group were needed.

### Statistical Analysis

The results are presented as the mean ± standard error (SEM) and were analyzed with GraphPad Prism (Version 7.0). The statistical significance of the long-term effects of drug treatments in TDS rats or IFS mice was determined using one-way analysis of variance (ANOVA), followed by Fisher’s LSD multiple comparison test. Statistical analyses between two groups (non-TDS + Veh rats vs. TDS + Veh rats; non-IFS + Veh mice vs. IFS + Veh mice) were performed by unpaired Student’s t-test. *p* < 0.05 was considered statistically significant in all tests. In addition, data loss caused by animal crawling out the experimental devices or animal death will not be included in the statistical analysis.

## Results

### Long-Term Behavioral Effects of YL-0919 in Post-TDS Rats

To determine whether the TDS procedure or drug administration could affect the baseline locomotor ability of rats, we conducted a spontaneous locomotor activity test in an open field. In terms of total distance moved, no significant differences were observed between the non-TDS rats and the post-TDS rats (t_20_ = 1.276, *p* > 0.05, Figure 1B). Daily oral administration of either Ser (15 mg/kg) or YL-0919 also did not significantly affect the total distance moved in the OFT (F_4, 45_ = 1.709, *p* > 0.05, Figure 1B).

In the NORT, RI represents the cognitive function, especially recognition memory, of the rats. As shown in Figure 1C, Student’s t-test revealed that TDS-exposed rats show significantly lower RI values than non-TDS rats (t_19_ = 4.067, *p* < 0.01). One-way ANOVA revealed that a significant effect for drugs treatment in terms of RI (F_4, 43_ = 3.295, *p* < 0.05). Post hoc analysis confirmed that treatment with YL-0919 (1.25, 2.5 mg/kg) significantly reversed the decrease in RI compared with vehicle treatment in the TDS rats (*p* < 0.05 and *p* < 0.01 for the dosages of 1.25 and 2.5, respectively). In contrast, repeated treatment with Ser (15 mg/kg) did not significantly affect the RI (*p* > 0.05).

The Y-maze test was conducted to evaluate the cognitive function associated with spatial working memory. As shown in Figure 1D, compared with the non-TDS rats, TDS-exposed rats showed significant reductions in the percentage of spontaneous alternations (t_19_ = 2.455, *p* < 0.05). We also found that repeated drugs treatment had a significant effect on the percentage of spontaneous alternations (One-way ANOVA, F_4, 44_ = 3.553, *p* < 0.05). Post hoc analysis confirmed that repeated treatment with YL-0919 (1.25, 2.5 mg/kg) resulted in a significant increase in the percentage of spontaneous alternations compared with the vehicle-treated TDS rats (*p* < 0.05 for the dosage of 1.25; *p* < 0.01 for the dosage of 2.5). Similar to the results in the NORT, repeated treatment with Ser (15 mg/kg) did not affect the decreased spontaneous alternations (*p* > 0.05). In addition, the TDS procedure or drug treatments did not significantly affect the total number of arms entries among groups (F_4, 44_ = 2.045, *p* > 0.05) (Figure 1E). The results from the spontaneous alternation test and NORT revealed that YL-0919 (1.25, 2.5 mg/kg) but not Ser had protective effects against the cognitive impairment induced by TDS in rats.

As shown in Figure 1F, rats exposed to the TDS procedure, compared with the non-TDS rats, showed a significant increase in the contextual freezing response (t_19_ = 11.06, *p* < 0.001, Figure 1F). Moreover, repeated drugs treatment showed main effects on the freezing responses in post-TDS rats (One-way ANOVA, F_4, 45_ = 11.44, *p* < 0.001). Further post hoc analysis confirmed that both chronic treatment with YL-0919 (1.25, 2.5 mg/kg) and Ser (15 mg/kg) significantly alleviated the enhanced contextual freezing responses compared with the vehicle-treated TDS rats (*p* < 0.001).

### Long-Term Behavioral Effects of YL-0919 in Post-Foot Shock Mice

The effects of chronic YL-0919 treatment on the freezing behavior of mice are shown in Figure 2B. Compared to the non-shocked mice, the mice exposed to the foot shocks showed a significantly increased freezing time as the contextual freezing measurement on day 7 (t_19_ = 8.951, *p* < 0.001). One-way ANOVA showed that drugs treatment induced significant suppression on the freezing responses (F_3, 37_ = 8.257, *p* < 0.001). Further post hoc analysis confirmed that both repeated treatment with YL-0919 (1.25, 2.5 mg/kg) and Ser (15 mg/kg) significantly reduced the freezing time compared with the TDS rats on day 7 (*p* < 0.001 for Ser and the dosage of 2.5 of YL-0919; *p* < 0.05 for the dosage of 1.25 of YL-0919). These results demonstrated that the mice had a sustained fear response to the ambient environment related to the traumatic events and indicated that chronic treatment with YL-0919 or Ser alleviated the contextual freezing behavior in the stressed mice, which were similar to the results observed in TDS rats.

The locomotor activity in each group in the OFT is shown in Figure 2C. The results revealed that there were no significant differences in the total distance moved among the groups, indicating that neither the aversive IFS procedure nor chronic drug treatments affected spontaneous locomotor activity of mice.

As Figures 2D,E show that on day 9 after exposure to foot shocks, the percentages of the open-arm duration time (t_17_ = 3.041, *p* < 0.01, Figure 2D) and the percentages of the open-arm entries (t_17_ = 3.265, *p* < 0.01, Figure 2E) were significantly decreased in vehicle-treated shocked mice compared to non-shocked mice, indicating that the shocked mice showed PTSD-associated anxiety-like behavior. One-way ANOVA showed that repeated drugs treatment caused a significant effect on the percentages of open-arm duration time (F_3,30_ = 8.671, *p* < 0.001, Figure 2D). Post hoc analysis confirmed that both repeated treatment with YL-0919 (2.5 mg/kg) and Ser (15 mg/kg) significantly reversed the decline of the percentages of open-arm duration time induced by IFS (*p* < 0.05 for YL-0919; *p* < 0.001 for Ser, Figure 2D). In addition, YL-0919 (1.25, 2.5 mg/kg) treatment exhibited a remarkable increase in the percentages of open-arm entries confirmed by post hoc analysis (*p* < 0.05, Figure 2E). A similar increasing tendency was produced by repeated treatment with Ser at the dose of 15 mg/kg (*p* = 0.06, Figure 2E).

The effects of YL-0919 on long-term recognition memory in IFS-exposed mice are depicted in Figure 2F. On day 12 after exposure to foot shocks, the RI values were significantly reduced in the IFS group (t_18_ = 2.166, *p* < 0.05). However, repeated treatment with Ser (15 mg/kg) resulted in a further and marked reduction in the RI confirmed by post hoc analysis (*p* < 0.05). By comparison, the results showed that repeated administration of YL-0919 (1.25 and 2.5 mg/kg) had no effect on the RI (*p* = 0.98 for the dosage of 1.25; *p* = 0.84 for the dosage of 2.5). These results indicated that YL-0919 compared to Ser treatment did not further aggravate stress-associated cognitive impairment induced by IFS in mice.

### Effects of YL-0919 on Synaptic Proteins and BDNF in the PFC

**FIGURE 3:**
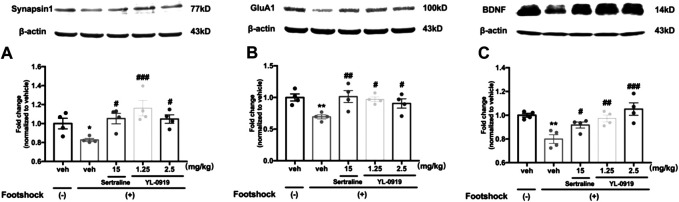
The effects of repeated administration with Ser or YL-0919 on expression levels of synaptic proteins and BDNF in the PFC in IFS-exposed mice. The post-IFS mice exhibit significant reductions in the expression levels of synapsin1 **(A)**, GluA1 **(B)** and BDNF **(C)**. YL-0919 (1.25 and 2.5 mg/kg) and sertraline (15 mg/kg) reverse the low expression levels of the three proteins. The levels of synapsin1, GluA1, and BDNF are shown as a ratio relative to *β*-actin. The data shown are the means ± SEM; *n* = 4; *p < 0.05, **p < 0.01 vs. the vehicle-treated foot-shock (−) group; ^#^ *p* < 0.05, ^##^ *p* < 0.01, ^###^ *p* < 0.001 vs. the vehicle-treated foot-shock (+) group.

The Western blotting results showed that the IFS procedure significantly decreased the levels of synaptic proteins (synapsin-1 and GluA1) and BDNF in the PFC compared with non-shocked mice (Figure 3A. synapsin-1, t_6_ = 2.962, *p* < 0.05; Figure 3B. GluA1, t_6_ = 4.78, *p* < 0.01; Figure 3C. BDNF, t_6_ = 4.981, *p* < 0.01). Compared with vehicle-treated shocked mice, repeated drugs treatment had a significant effect on the expression levels of above proteins (one-way ANOVA, Figure 3A synapsin-1, F_3,12_ = 6.683, *p* < 0.01; Figure 3B. GluA1, F_3,12_ = 4.786, *p* < 0.05; Figure 3C. BDNF, F_3,12_ = 8.041, *p* < 0.01). Post hoc analysis confirmed that mice receiving repeated administration of YL-0919 (1.25 and 2.5 mg/kg) showed a significant reversal of the reductions in these proteins (synapsin-1, *p* < 0.001 and *p* < 0.05 for the dosages of 1.25 and 2.5, respectively; GluA1, *p* < 0.05 for the dosages of 1.25 and 2.5; BDNF, *p* < 0.01 and *p* < 0.001 for the dosages of 1.25 and 2.5, respectively). A similar result was obtained after Ser treatment (synapsin-1, *p* < 0.05; GluA1, *p* < 0.01; BDNF, *p* < 0.05). These results suggested that YL-0919 alleviated the foot shock-induced PTSD-like behaviors, which might be mediated by upregulating the expression of BDNF and the formation of synaptic proteins in the PFC.

### Effects of YL-0919 on Dendritic Complexity and Spine Density in the PFC

**FIGURE 4:**
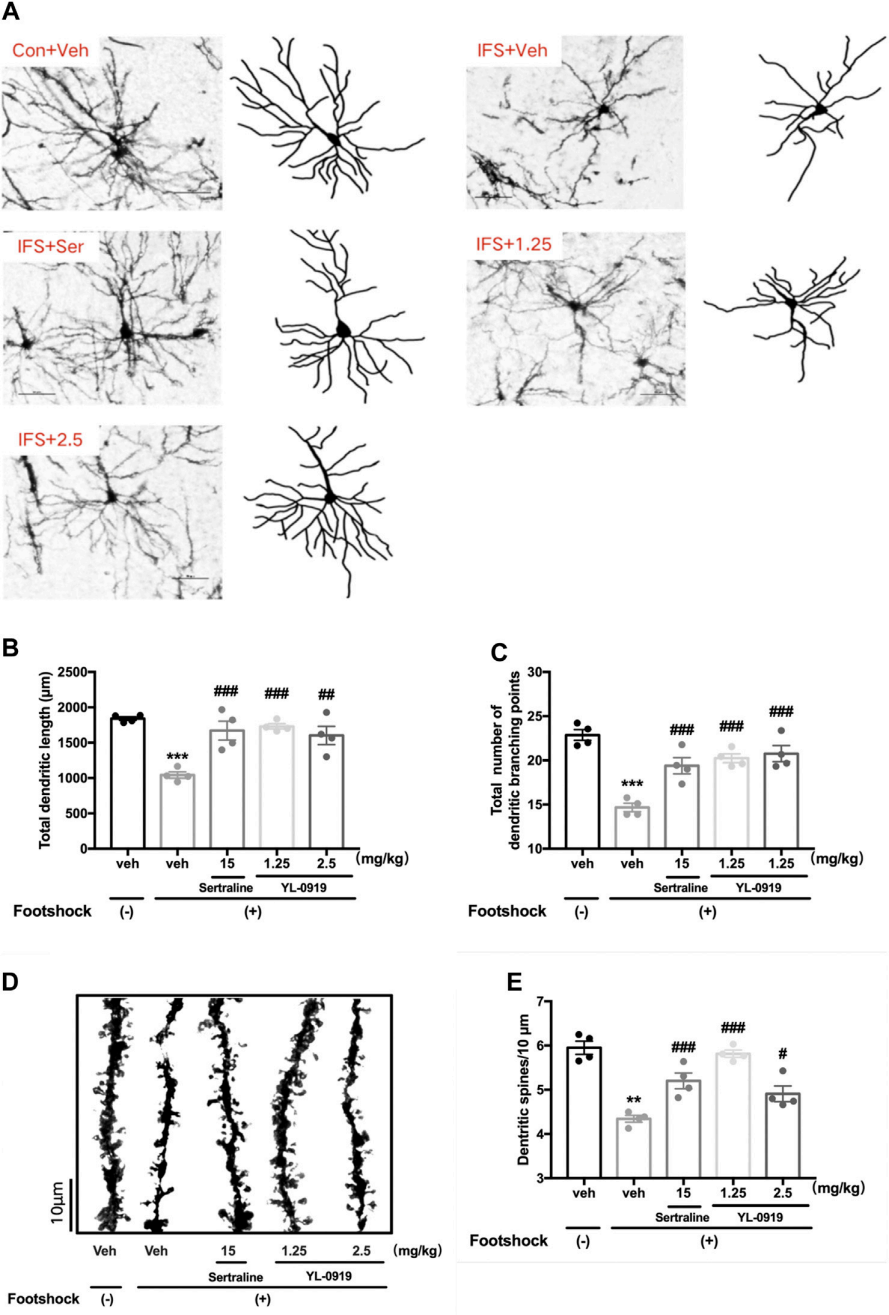
The effects of repeated administration with Ser or YL-0919 on dendritic complexity and spine density of PFC pyramidal neurons in IFS-exposed mice. **(A)** Typical diagram of the Golgi-impregnated pyramidal neurons and Neuron J tracking in the region of the PFC from each group. Scale bar = 50 μm. **(B, C)** Similar to the repeated treatment with Ser (15 mg/kg), repeated treatment with YL-0919 (1.25 and 2.5 mg/kg) significantly reversed the reductions in the total dendritic length and the total number of dendritic branches of the pyramidal neurons induced by the IFS procedure. **(D)** Representative photomicrograph of dendritic spines from each group. Scale bar = 10 μm. **(E)** Similar to the effect of Ser (15 mg/kg) treatment, the decreased spine density induced by IFS was significantly reversed by YL-0919 (1.25 and 2.5 mg/kg) treatment. The data shown are the means ± SEM; *n* = 4; **p < 0.01, ***p < 0.001 vs. the vehicle-treated foot-shock (−) group; ^#^ *p* < 0.05, ^##^ *p* < 0.01, ^###^ *p* < 0.001 vs. the vehicle-treated foot-shock (+) group.

The morphology of pyramidal neurons in the PFC was observed by Golgi staining. The typical patterns of the changes in dendritic complexity are shown in Figure 4A. The results revealed significant deficits in the total dendritic length (t_6_ = 15.28, *p* < 0.001, Figure 4B) and the total branching points of dendrites (t_6_ = 10.63, *p* < 0.001, Figure 4C) in the PFC from mice subjected to the IFS procedure compared with mice in the control group. Compared with vehicle administration, one-way ANOVA revealed a significant effect for drugs treatment (Figure 4B total dendritic length, F_3,12_ = 10.59, *p* < 0.01; Figure 4C total branching points of dendrites, F_3,12_ = 14.45, *p* < 0.001). Post hoc analysis confirmed that repeated treatment of YL-0919 (1.25 and 2.5 mg/kg) significantly increased dendritic complexity (Figure 4B total dendritic length, *p* < 0.001 and *p* < 0.01 for the dosages of 1.25 and 2.5, respectively; Figure 4C total branching points, *p* < 0.001 for the dosages of 1.25 and 2.5). These effects were similar to those with Ser administration, which resulted in increased dendritic length and branching points (*p* < 0.001).

Representative diagrams of dendritic spines in the different groups are shown in Figure 4D. A marked reduction in dendritic spine density in the PFC was observed in the IFS group (t_6_ = 4.451, *p* < 0.01, Figure 4E) compared with the control group, which was dramatically reversed by drugs treatment (F_3, 12_ = 19.98, *p* < 0.001, one-way ANOVA). Post hoc analysis confirmed that repeated treatment of YL-0919 (1.25 and 2.5 mg/kg) significantly increased spine density compared with the vehicle-treated IFS group (*p* < 0.001 and *p* < 0.05 for the dosages of 1.25 and 2.5, respectively). These effects were similar to those in the Ser (15 mg/kg) treatment group (*p* < 0.001). The above results suggested that ameliorating the disruptions in dendritic complexity and spine density in the PFC might be involved in the anti-PTSD behavioral effect of YL-0919.

## Discussion

In the present study, we found that chronic YL-0919 treatment caused significant suppression in the contextual fear, enhanced anxiety and cognitive dysfunction induced by the inescapable electric foot-shock in a mouse model of PTSD and by the TDS procedure in rats. In addition, the anti-PTSD effects of YL-0919 might be partially mediated by ameliorating structural neuroplasticity by increasing the expression of BDNF and the formation of synapse-related proteins in the PFC.

Previous studies have demonstrated that animals exposed to severe and different stressors (e.g., restraint, forced swim and ether anesthesia) and situational reminders (e.g., re-exposure to forced swim) of traumatic events in TDS procedure showed lasting PTSD-like symptoms, which suggested that TDS was a proven PTSD animal model that could mimic the long-term behavioral and neuroendocrine alterations observed in human patients (Richter-Levin et al., 2019). In the current study, the data showed that TDS exposure induced significant increases in freezing time in the contextual fear paradigm. Repeated treatment with YL-0919 successfully reversed the enhanced contextual fear response, which indicated that YL-0919 treatment showed a therapeutic effect in this animal model within a certain dosage range, and a similar effect was observed after Ser treatment. Given the cognitive decline in patients with PTSD and the role of 5-HT_6_ receptors in improving cognitive function, we then investigated the effects of YL-0919 on PTSD-associated impairments in cognitive function in the TDS rats. Our results showed that TDS-exposed rats exhibited considerable memory impairment, including recognition memory and spatial working memory, as evidenced by the reductions in the RI and percentage of spontaneous alternations in the NORT and Y-maze test, respectively. The results were in accordance with previous reports that a single prolonged stress (SPS) PTSD model in rats exhibited social and object recognition memory deficits in the social choice test (SCT) and NORT 7 days after stress (Eagle et al., 2013). Our data demonstrated that repeated treatment with YL-0919 (1.25–2.5 mg/kg), but not Ser, significantly ameliorated memory recall in the NORT and alternation performance in the Y-maze test.

To further investigate the anti-PTSD effects of YL-0919, the IFS model was used. Numerous studies have shown that the IFS model is a well-validated PTSD animal model that generates long-term PTSD-like symptoms commonly observed in PTSD patients, such as intrusive memories, avoidance, defensive behavior and hyperarousal (Homberg, 2015). In this study, a 2-days regimen of foot shocks significantly enhanced contextual fear behavior, which was consistent with our previous findings (Qiu et al., 2013). Repeated YL-0919 treatments significantly decreased freezing time, indicating that YL-0919 alleviated the fear response of stressed mice to the aversive environment. In addition, YL-0919 reversed the anxiety-like behavioral changes induced by the aversive IFS procedure, as manifested by increasing the percentages of open-arm durations and the percentages of open-arm entries, indicating the alleviating effect of YL-0919 on PTSD-associated anxiety-like behaviors.

Furthermore, the experiments performed in mice also showed that IFS exposure caused a clear impairment in recognition memory in the NORT, as manifested by the decrease in the RI. In contrast, unlike Ser treatment, repeated YL-0919 treatment did not enhance the IFS-induced decline in the RI, which demonstrated that YL-0919, compared to Ser, did not further aggravate the impaired cognitive function in IFS-exposed mice. Repeated paroxetine (another first-line SSRI drug for PTSD treatment) administration (20 mg/kg, i. p.) for 15 days significantly impaired the short- and long-term memory of normal rats in the radial arm maze test (Menezes et al., 2018), which was similar to the negative effects of Ser on cognitive performance in the current study. More cognitive behavioral paradigms are needed to evaluate the effects of Ser or YL-0919 on cognitive function in mouse model of PTSD induced by IFS.

It was reported that vilazodone, acting as an SSRI and 5-HT_1A_ receptor partial agonist, had no effect on cognitive function (Li et al., 2017). However, our previous study found that sub-chronic treatment with YL-0919 had a considerable memory-enhancing effect in the NORT, and direct activation of 5-HT_6_ receptors partially mediated this effect (Chen et al., 2018). Interestingly, 5-HT_6_ receptor antagonists have also been shown to enhance cognitive performance in different learning and memory tests performed on rodents and primates, and antagonism of the 5-HT6 receptor is a promising approach for the symptomatic treatment of Alzheimer’s disease (AD) (Foley et al., 2004; Upton et al., 2008; de Jong and Mørk, 2017). Given the target differences among YL-0919, Ser and vilazodone, it is possible that 5-HT_6_ receptors might be involved in the pharmacological effects of YL-0919 on cognitive function. In addition, YL-0919 showed different effects on PTSD-associated cognitive impairment in the present study. One possibility is that there is a differential sensitivity to drugs in the different animal models. In addition, strains of rodents should also be taken into account.

It should be noted that although the animals exposed to different and severe stressors showed PTSD-like behaviors (freezing, anxiety-like behavior and cognitive impairment), the results in the locomotor activity test showed that neither the aversive TDS/IFS procedure nor repeated drug treatments significantly affected the total distance moved by the animals, indicating that the occurrence and amelioration of PTSD-like behaviors were not caused by influences on the locomotor activity, which are related to physical condition.

Taken together, the *in vivo* results observed in this study indicated anti-PTSD effects of YL-0919 at the doses tested, which warrants a further pharmacological characterization of this compound. Alterations in neuronal plasticity are involved in many psychiatric disorders, while functional and structural alterations of dendritic spines are crucial for synaptic plasticity. To investigate the possible molecular mechanisms underlying the anti-PTSD effects of YL-0919, we then examined the expression levels of BDNF and synaptic proteins and changes in neuronal structure plasticity, including dendritic complexity and dendritic spine density, in the PFC in mice.

Over the past decade, BDNF has been shown to regulate stress- and anxiety-related behaviors while playing a critical role in the fear circuit. A considerable number of studies have revealed the connections between the BDNF (Val66Met) polymorphism and both the occurrence risk and symptom severity of PTSD. And BDNF^Met/Met^ genotype has been proven to induce defects in extinction learning in mice (Notaras and van den Buuse, 2020). Furthermore, an accumulating number of studies have shown that the BDNF can not only maintain the structure of dendritic trees, especially in the processes of refinement and stabilization, but also modify the shape and number of dendritic spines (Gorski et al., 2003; Gao et al., 2009). The current findings showed that YL-0919 administration could significantly relieve the decreased expression level of BDNF in IFS mice. These results were in accordance with our previous study in which the level of BDNF was significantly reduced in the TDS rat model (Zhang et al., 2015).

Dendritic spines are extremely plastic, and their number as well as shape can be changed in relatively short periods of time based on different stimuli (Gipson and Olive, 2017). Synaptic function is affected when the density of dendritic spines changes, and the number of spines are involved in neuronal connectivity and excitability (Yuste, 2013). Moreover, alterations in the morphology of dendrites could impact the amount of synaptic inputs and disrupt functional integrity of the mPFC in stressed mice (Izquierdo et al., 2006). In our study, IFS exposure contributed to the long-term disruptions of the morphological plasticity in the PFC, as evidenced by the decreased dendritic complexity and spine density. This findings were consistent with studies in which acute stress caused impairments in neuronal plasticity (Izquierdo et al., 2006). Therefore, the abnormal morphological changes might be associated with PTSD-like behaviors, as well as the decline in object recognition memory and the decreased levels of synaptic proteins. We also showed that the impaired neuroplasticity and the decreased levels of synaptic proteins in the PFC were reversed by repeated YL-0919 treatment. It has been well established that the synthesis of synaptic proteins are important for long-term synaptic plasticity (Costa-Mattioli et al., 2009). The rapid translation of synaptic proteins such as synapsin1, PSD-95 (Lee et al., 2005) and GluA1 (Schratt et al., 2004) can be regulated with the activation of mTOR signaling. In addition, it has been reported that the expression of plasticity-associated genes (e.g., those related to mTOR signaling) is upregulated in the frontal cortex due to the 5-HT receptor activities (du Jardin et al., 2016), which is consistent with the BDNF-mTOR signaling activation by YL-0919 in our previous study (Sun et al., 2019). Thus, the increased levels of BDNF followed by modulation of neuronal plasticity in the PFC may be involved in the anti-PTSD effects of YL-0919.

Although the amelioration of synaptic morphological plasticity by YL-0919 treatment was observed in this study, and LTP in the hippocampus was enhanced by 7-days YL-0919 treatment in our previous study (Zhang et al., 2017), further electrophysiological determinations are needed to assess the functional changes in synaptic plasticity after exposure to the IFS/TDS procedure and YL-0919 treatment.

It should be noted that, in our present study, it is difficult to distinguish between sub-chronic or chronic effects of YL-0919 due to experiment design of TDS rats. Some of the reasons may lead to this result, such as the long period for TDS modeling, the large number of behavioral experiments, and the long duration of some experiments. Different behavioral experiments could be performed in other separate cohorts on the same point to ensure the consistency of drug effects.

In conclusion, our study revealed that YL-0919, a triple-target compound acting as an SSRI, a 5-HT_1A_ receptor partial agonist and a 5-HT_6_ receptor full agonist, exerted a significant anti-PTSD effects partially mediated by ameliorating the structural neuroplasticity by increasing the expression of BDNF and the formation of synaptic proteins in the PFC. Our results provide a preclinical basis for the application of novel antidepressants in PTSD treatment, especially in patients with PTSD-induced cognitive impairment.

## Data Availability

The raw data supporting the conclusion of this article will be made available by the authors, without undue reservation.
